# Integrating multi-omics with machine learning: from genomic variant functional annotation to pharmacogenomics and biomarker discovery

**DOI:** 10.3389/fphar.2026.1980359

**Published:** 2026-10-14

**Authors:** Jing Lv, Jinjin Guo, Yangyang Wang, Jihan Wang, Lei Guo, Hongxing Zhang

**Affiliations:** 1 Department of Clinical Laboratory, Honghui Hospital, Xi’an Jiaotong University, Xi’an, Shaanxi Province, China; 2 Department of Hand Surgery, Honghui Hospital, Xi’an Jiaotong University, Xi’an, Shaanxi Province, China; 3 School of Physics and Electronic Information, Yan’an University, Yan’an, Shaanxi Province, China; 4 Yan’an Medical College, Yan’an University, Yan’an, Shaanxi Province, China; 5 Department of Spine Surgery, Honghui Hospital, Xi’an Jiaotong University, Xi’an, Shaanxi Province, China

**Keywords:** machine learning, multi-omics integration, personalized medicine, pharmacogenomics, precision oncology, variant interpretation, variants of uncertain significance (VUS)

## Abstract

Integrating heterogeneous multi-omics data with machine learning (ML) provides a powerful framework for interpreting genomic variants and advancing pharmacogenomic research in precision medicine. High-throughput technologies have generated vast datasets spanning genomics, transcriptomics, epigenomics, and proteomics, yet integrating these high-dimensional data into clinically meaningful insights remains a major computational challenge. Machine learning and deep learning models, including Graph Neural Networks (GNNs), Transformers, and Variational Autoencoders (VAEs), offer promising solutions. This narrative review provides a state-of-the-art overview of ML-driven multi-omics integration across early, intermediate, and late fusion paradigms. We highlight how these approaches may support functional variant interpretation, including the reclassification of Variants of Uncertain Significance (VUS), and emerging pharmacogenomic applications. Key areas include predictive biomarkers for drug efficacy, therapeutic resistance in precision oncology, and personalized dosing in rare and complex diseases. Finally, we discuss challenges related to data heterogeneity, clinical interpretability, and regulatory approval, as well as the requirements for translating emerging computational approaches into clinically validated pharmacogenomic practice and precision pharmacotherapy.

## 1 Introduction

The past two decades have witnessed a paradigm shift in human genetics, driven by the precipitous drop in sequencing costs and the widespread adoption of Next-Generation Sequencing (NGS) technologies (Evans, 2025; Ng et al., 2025). From the completion of the Human Genome Project to the massive datasets generated by initiatives such as the UK Biobank and the All of Us Research Program, our capacity to catalogue human genetic variation has reached an unprecedented scale (Gouveia et al., 2025; Yang Y. et al., 2026). Genome-wide association studies (GWAS) and whole-exome/genome sequencing (WES/WGS) have successfully identified millions of variants associated with complex traits and rare diseases (Pathan et al., 2024). Today, the fundamental challenge is no longer the acquisition of genetic data, but rather its functional and clinical interpretation, as a critical “genotype-phenotype gap” persists despite the genomic deluge (Li J. et al., 2026).

In hereditary and Mendelian disease testing, a substantial proportion of detected germline variants remain functionally uncharacterized and are therefore classified as variants of uncertain significance (VUS) (Horowitz et al., 2025; Chen et al., 2023). This classification creates a clinical impasse because the pathogenicity and phenotypic consequences of such variants cannot be determined with sufficient confidence for clinical decision-making (Peyser et al., 2025). Furthermore, the “missing heritability” problem suggests that static genomic variants—acting in isolation—cannot fully explain the heterogeneity observed in disease onset, progression, and drug response (Montesinos-López et al., 2025). The static nature of the DNA sequence fails to capture the dynamic, tissue-specific, and environmental interactions that ultimately dictate cellular function and disease states (Xiong et al., 2025). To bridge this gap, the field is moving beyond a strictly “gene-centric” view toward a “systems biology” approach (Yang Y. et al., 2025). This involves integrating genomics with downstream “omics” layers, including epigenomics (regulation), transcriptomics (expression), proteomics (translation), and metabolomics (function) (Yang J. et al., 2026). These layers serve as quantifiable intermediate phenotypes, providing the necessary functional context to interpret genetic variation dynamically (Tokolyi et al., 2025).

Within clinical variant interpretation frameworks such as the ACMG/AMP guidelines, multi-omics data may contribute functional evidence relevant to PS3/BS3 classification. However, such observations do not automatically satisfy PS3/BS3 criteria; the strength of the evidence depends on the analytical and clinical validation of the assay, its biological relevance to the disease mechanism, the use of appropriate controls, and any applicable disease- or gene-specific specifications (Ma et al., 2026). For example, evidence that a non-coding variant alters chromatin accessibility or induces aberrant splicing may support variant interpretation when generated using a sufficiently validated and biologically relevant assay (Stark et al., 2025). By capturing the flow of biological information, a single genetic variant’s effect can be translated into a multi-omic signature, thereby evolving from a static genetic marker into a dynamic, clinically actionable biomarker (Hsu et al., 2025). However, capitalizing on this “variant-to-biomarker” pipeline presents a formidable computational challenge. Multi-omics datasets are characterized by massive high dimensionality, inherent heterogeneity, technical noise, and complex non-linear biological interactions that traditional statistical methods struggle to model effectively (Li A. Z. et al., 2025; Spooner et al., 2025). Consequently, Artificial Intelligence (AI) and Machine Learning (ML) have emerged as important computational tools. Modern ML architectures, particularly Deep Learning (DL), can learn latent representations from diverse data modalities, identifying subtle multi-omic patterns that escape human analysis, and predicting clinical outcomes with high accuracy (Elumalai and Surendran, 2025; Song W. et al., 2026).

This review provides a narrative overview of the intersection of human genetics, multi-omics integration, and artificial intelligence. We examine current strategies for bridging the genotype-phenotype gap, focusing exclusively on how ML models integrate diverse omics layers to interpret human genetic variation. Specifically, we discuss methodological frameworks for data integration, evaluate state-of-the-art ML algorithms utilized in biomarker discovery, and highlight successful applications in clinical translation—ranging from functionally reclassifying VUS in rare diseases to advancing precision oncology. As outlined in Figure 1, the architecture of this review mirrors the translational pipeline: beginning with the multi-omics biological landscape, progressing through computational data fusion and machine learning algorithms, and culminating in real-world clinical applications and future perspectives.

**FIGURE 1:**
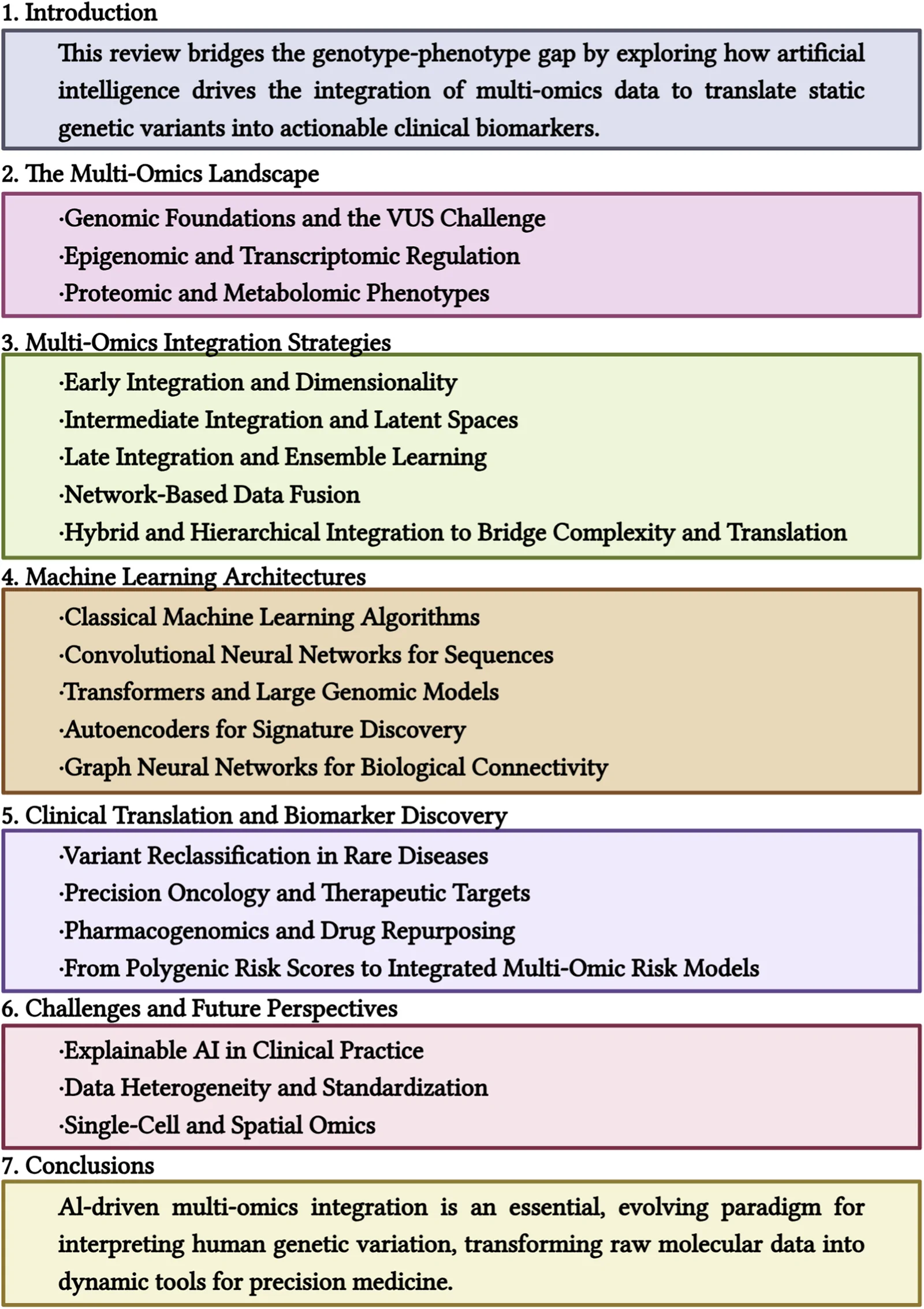
Visual outline of the review. This review explores the transition from static genetic variants to dynamic clinical biomarkers. The workflow highlights the biological multi-omics landscape (Section 2), fundamental data fusion strategies (Section 3), state-of-the-art machine learning architectures (Section 4), and their translational applications across diverse clinical domains (Section 5), concluding with current challenges and future multi-omics resolutions (Section 6).

## 2 The multi-omics landscape

The traditional linear central dogma is insufficient for interpreting the full complexity of human genetic variation. Instead, translating a static variant into an actionable biomarker requires navigating an interconnected multi-omics landscape (van Hilten et al., 2024). Understanding the functional context of these diverse molecular layers is a crucial prerequisite for leveraging machine learning to bridge the genotype-phenotype gap (Benachour et al., 2026).

### 2.1 Genomic foundations and the VUS challenge

Genomics serves as the foundational layer of biological information, cataloging the innate genetic blueprint (Pagnamenta et al., 2023). Over the past decade, advances in whole-exome and whole-genome sequencing (WES/WGS) have democratized the discovery of single nucleotide variants (SNVs), insertions/deletions (indels), and structural variants across diverse populations (Lee J. K. et al., 2025; X et al., 2025). In this section, the VUS discussion primarily refers to germline variants encountered in Mendelian and hereditary disease testing; somatic variants arising in cancer are considered separately in the precision oncology section because their interpretation and clinical implications differ. However, this diagnostic triumph has simultaneously precipitated a severe interpretive crisis. As sequence data accumulates in repositories like ClinVar, the overwhelming majority of newly discovered sequence alterations are classified as VUS (Folta et al., 2025).

The VUS challenge fundamentally stems from a lack of definitive functional evidence. While classical *in silico* tools relying on evolutionary conservation or protein structure predictions (e.g., CADD, REVEL, PolyPhen) offer preliminary pathogenic scores, they frequently lack the specificity required to alter clinical management (Heo and Kim, 2025; Gudkov et al., 2025). Under stringent diagnostic frameworks, such as the ACMG/AMP variant interpretation guidelines, a genomic sequence alone provides the coordinate of a mutation but rarely elucidates its mechanistic consequence (Rius et al., 2025). This is particularly problematic for non-coding variants, synonymous mutations, or deep intronic alterations where the impact on the final protein product is not intuitively obvious. Overcoming this genomic bottleneck necessitates moving beyond the static DNA sequence to observe downstream functional effects directly (Pitsava et al., 2025).

### 2.2 Epigenomic and transcriptomic regulation

To understand how a genomic variant perturbs cellular function, the immediate downstream regulatory and expressive layers must be interrogated. The epigenome acts as the master regulatory switch, determining genome accessibility and higher-order chromatin architecture. Assays such as ATAC-seq (chromatin accessibility) and whole-genome bisulfite sequencing (DNA methylation) reveal how genetic variants disrupt regulatory elements like enhancers and promoters (Dudek et al., 2025; Arthur et al., 2025). These perturbations, often characterized as methylation or chromatin quantitative trait loci (QTLs), provide critical mechanistic evidence for non-coding variants that would otherwise remain uninterpretable (Matos et al., 2026).

Downstream of epigenetic regulation, transcriptomics captures the dynamic messenger RNA (mRNA) landscape. RNA sequencing (RNA-seq) has emerged as a powerful adjunct to DNA sequencing in clinical diagnostics (Lewis et al., 2025). It directly quantifies the immediate functional consequences of genetic variants, most notably through the detection of aberrant splicing events (such as exon skipping, intron retention, or cryptic splice site activation), allele-specific expression (ASE, indicating nonsense-mediated decay or imprinting defects), and overall gene dysregulation (Silv et al., 2026). By serving as a quantifiable intermediate phenotype, the transcriptome bridges the initial gap between the genomic blueprint and cellular behavior, translating predicted variant effects into observable molecular aberrations (Renganaath and Albert, 2025; Chen et al., 2025a).

### 2.3 Proteomic and metabolomic phenotypes

While transcriptomics offers robust evidence of gene expression, mRNA levels do not universally correlate with ultimate protein abundance or function due to extensive post-transcriptional buffering and degradation mechanisms. Proteomics, therefore, represents a crucial step closer to the true functional phenotype (Li et al., 2026b). Advanced mass spectrometry and high-throughput affinity-based assays map not only protein abundance but also critical post-translational modifications (PTMs), protein isoforms, and protein-protein interaction (PPI) networks. A genetic variant may not significantly alter mRNA levels but could destabilize the folded protein or disrupt its interaction interface—pathogenic effects that only proteomic profiling can capture (Sharma et al., 2025).

Metabolomics provides the ultimate snapshot of the cellular microenvironment and physiological state. Metabolites are the downstream products of genomic, transcriptomic, and proteomic cascades, influenced simultaneously by the microbiome and environmental exposures (Wang J. et al., 2025). Measuring metabolic flux or specific metabolite concentrations offers a highly sensitive, real-time readout of biological disruption (Zhang et al., 2025a). In the context of the “variant-to-biomarker” pipeline, the proteome and metabolome transform the abstract genetic risk identified in the genome into tangible, circulating molecules. These functional layers frequently harbor the most direct indicators of disease pathology, serving as the definitive molecular biomarkers required for clinical diagnosis, prognosis, and therapeutic targeting (Ren et al., 2025; Yuan et al., 2026). However, integrating these disparate, high-dimensional layers to extract a cohesive biological signal exceeds human analytical capacity, mandating the deployment of advanced machine learning architectures (Narayanan et al., 2025). Figure 2 depicts the multi-omic central dogma pyramid, which outlines the hierarchical organization of molecular layers in the cell. The genome provides the static genetic sequence, which is regulated by the epigenome to control gene expression. The resulting transcriptome is translated into the functional proteome, which ultimately shapes the metabolome, the end product of cellular metabolism. The unique diagnostic utility and inherently heterogeneous computational data structures of each omics layer are summarized in Table 1, highlighting the multidimensional complexity that machine learning architectures must navigate to discover actionable biomarkers.

**FIGURE 2:**
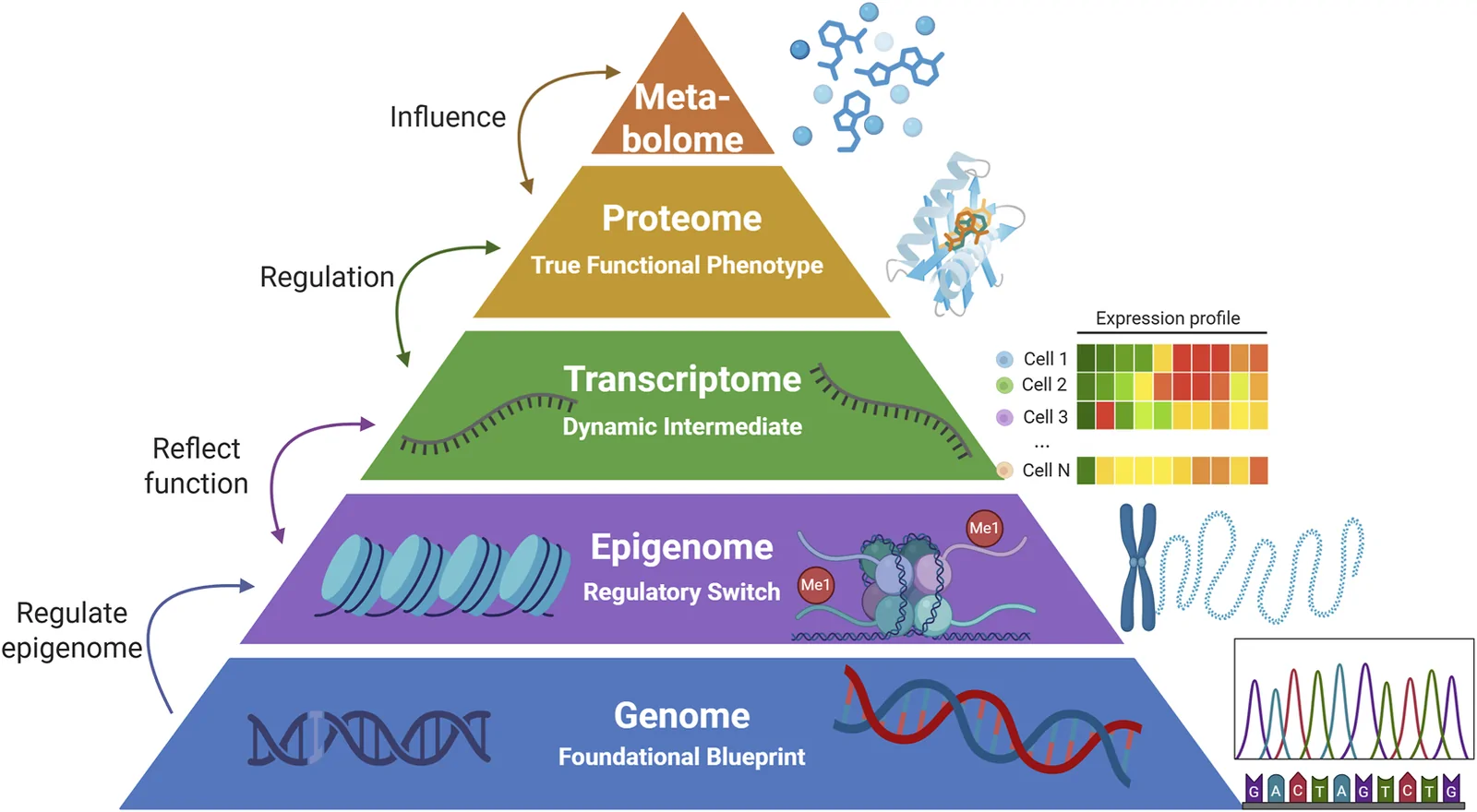
The multi-omic central dogma pyramid: hierarchical organization and regulatory relationships across genomic, epigenomic, transcriptomic, proteomic, and metabolomic layers. The foundational layer is the genome, the static DNA sequence that serves as the organism’s genetic blueprint. Above it, the epigenome acts as a regulatory switch, modulating chromatin accessibility and gene expression via modifications such as histone methylation (Me1) without altering the underlying DNA sequence. The transcriptome, a dynamic intermediate, reflects active gene expression through mRNA profiles, which are further translated into the proteome—the true functional phenotype of the cell, composed of folded proteins and protein complexes. At the apex, the metabolome represents the end products of cellular processes, influenced by upstream proteomic activity. Arrows denote directional regulatory relationships: the genome shapes the epigenome, which in turn regulates the transcriptome; the transcriptome drives the proteome, which ultimately influences the metabolome, while each layer also reflects functional states of the system.

**TABLE 1:** Characteristics of multi-omics layers for variant interpretation and biomarker discovery.

Omics layer	Primary technologies	Molecular signatures detected	Contribution to variant interpretation	Computational data structure
Genomics	WGS, WES, Targeted Gene Panels	SNVs, Indels, CNVs, Structural Variants (SVs), Loss of Heterozygosity (LOH)	Foundational: Provides variant, population-frequency, and consequence information that may contribute to ACMG/AMP assessment when criterion-specific requirements are met.	1D Character strings (A, T, C, G); Categorical matrices (VCFs); Variant graphs
Epigenomics	ATAC-seq, ChIP-seq, WGBS	Chromatin accessibility, Histone modifications, DNA methylation states (e.g., mQTLs)	Regulatory Evidence: Provides mechanistic context for non-coding variants and may complement computational evidence of regulatory disruption	1D Continuous signal tracks (e.g., bedGraph); Sparse count matrices
Transcriptomics	Bulk RNA-seq, Isoform sequencing (Iso-Seq), scRNA-seq	Aberrant splicing (exon skipping, intron retention), Allele-specific expression (ASE), Gene dysregulation	Functional Evidence: Provides evidence of gene/transcript disruption and may contribute to PS3/BS3 assessment when supported by a validated, biologically relevant assay with appropriate controls	High-dimensional continuous count matrices; Sashimi plots (1D structural graphs)
Proteomics	Mass Spectrometry (LC-MS/MS), Affinity-based assays (Olink, SomaScan)	Protein abundance, post-translational modifications (PTMs), Protein isoforms, Protein-protein interactions (PPIs)	Functional Evidence: Can demonstrate altered protein abundance, stability, or interactions and may support functional evidence assessment in an appropriate gene- and disease-specific context	Continuous intensity matrices; 3D spatial coordinate graphs; Undirected topological graphs (PPI networks)
Metabolomics	NMR Spectroscopy, Untargeted/Targeted Mass Spectrometry	Metabolic flux, small molecule concentrations, Lipidomic profiles	Biochemical Evidence: Provides downstream metabolic evidence that may support variant interpretation, depending on assay validity, biological specificity, and disease context	High-dimensional continuous variables; Directed metabolic pathway graphs

## 3 Multi-omics integration strategies

Multi-omics data exhibits extreme heterogeneity, spanning discrete genomic variables to continuous transcriptomic and proteomic matrices. Integrating these disparate modalities is a crucial computational prerequisite, as the chosen fusion strategy profoundly impacts downstream machine learning performance in biomarker translation (Spooner et al., 2025; Mukhdoomi and Chachoo, 2025). These computational paradigms are broadly categorized into early, intermediate, late, network-based, and hybrid integration. Figure 3 visually summarizes these foundational architectures, illustrating how they balance biological interpretability with clinical deployment constraints prior to AI modeling.

**FIGURE 3:**
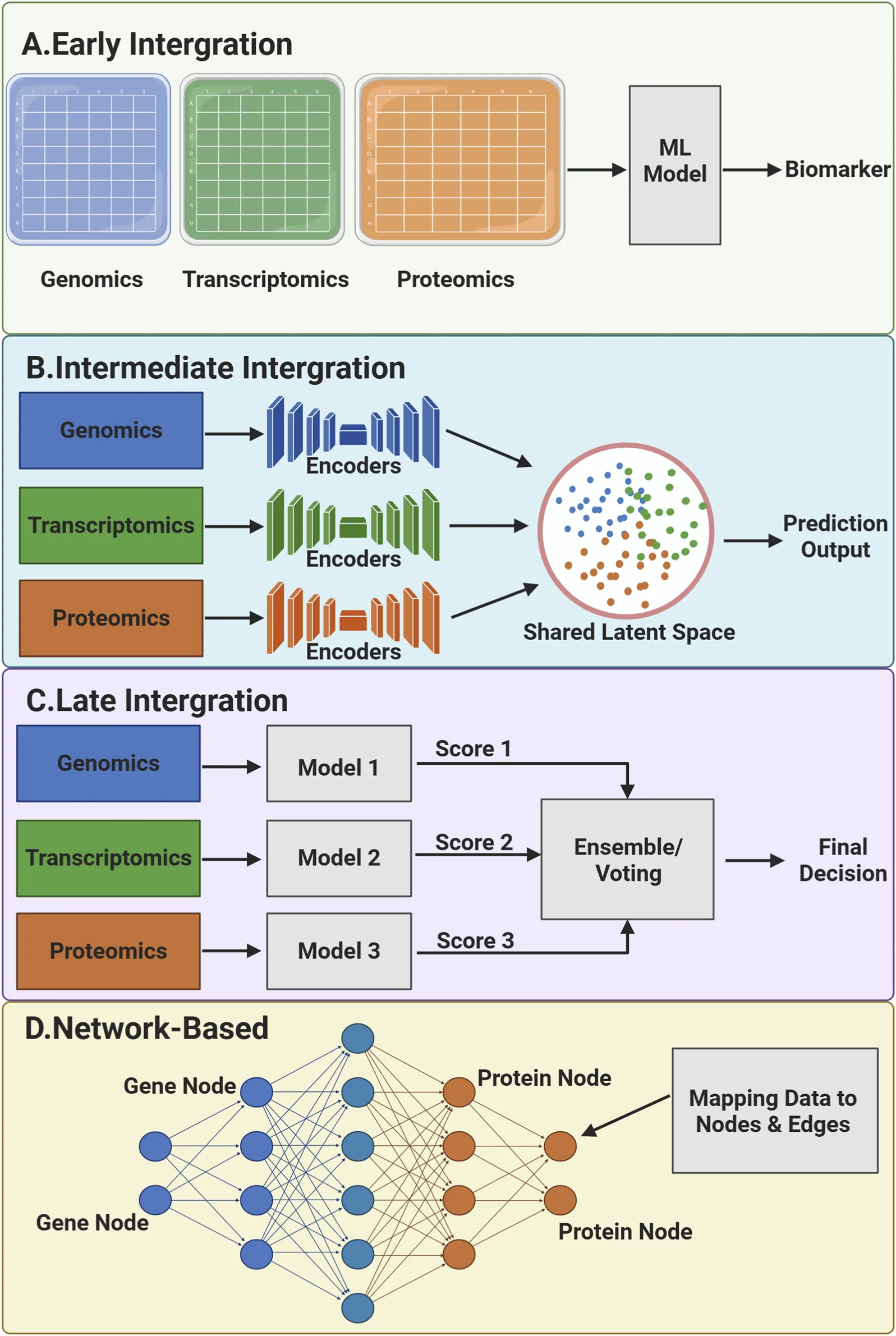
Architectural paradigms of multi-omics integration strategies for variant-to-biomarker translation. **(A)** Early Integration: Features from distinct omics layers are concatenated into a single matrix prior to ML training. The resulting high-dimensional space (p ≥ n) risks overfitting, leading to low clinical translation readiness. **(B)** Intermediate Integration: Independent modalities are processed via encoders into a lower-dimensional “Shared Latent Space,” capturing joint biological variance to extract composite signatures (e.g., for VUS identification). **(C)** Late Integration: Modality-specific models are trained independently to generate separate scores, which are aggregated via ensemble learning. This modular approach can accommodate missing modalities more flexibly than approaches requiring complete multi-omics profiles, although its practical performance and clinical applicability depend on the dataset, validation strategy, and implementation context. **(D)** Network-Based Integration: Multi-omics profiles are superimposed onto biological graphs, where quantitative data dictates node attributes and interactions constitute edges, forming a topological foundation for advanced modeling. Abbreviations: ML, Machine Learning; VUS, Variant of Uncertain Significance.

### 3.1 Early integration and dimensionality

Early integration, frequently referred to as data-level fusion, is the most intuitive computational approach. It involves concatenating the primary features from multiple omics layers into a single, unified data matrix before model training (Sun et al., 2026). For a given clinical cohort, a patient’s genetic variants, RNA expression counts, and protein abundance levels are horizontally merged into one expansive vector (Zhang D. et al., 2025). This comprehensive input is then fed directly into standard machine learning algorithms. The primary advantage of early integration is its ability to capture holistic cross-modality interactions from the very beginning of the analytical pipeline (Yan et al., 2025; Chen K. et al., 2025).

However, this approach immediately encounters the “curse of dimensionality,” a pervasive challenge in human genetics where the number of biological features typically dwarfs the number of available clinical samples (the *p*≫*n* problem) (Lan et al., 2025). Concatenating millions of variants with tens of thousands of transcripts exponentially expands the feature space, leading to severe model overfitting and computational inefficiency (Peng, 2025; Bukovsky et al., 2025). Furthermore, early integration is susceptible to modality dominance, where high-dimensional data (e.g., genome-wide methylation arrays) overshadows narrower but highly informative functional data (e.g., targeted proteomics) (Wu et al., 2025a). Consequently, robust dimensionality reduction and rigorous feature selection are mandatory pre-processing steps to distill actionable biomarker signals from the concatenated noise (Zheng et al., 2025). Importantly, to prevent data leakage and overly optimistic performance estimates, preprocessing steps such as normalization, imputation, dimensionality reduction, and feature selection should be fitted exclusively within the training folds and then applied to the corresponding validation or test data. From a practical standpoint, while early integration is invaluable for baseline exploratory research, its “clinical translation readiness” is notoriously low (Ye et al., 2025). As frequently noted in recent computational oncology reviews, deploying an early integration model in a real-world hospital requires all omics assays (e.g., WGS, RNA-seq, and mass spectrometry) to be completed simultaneously for a single prediction. This high diagnostic cost heavily restricts its transition from bench to bedside (Meng et al., 2024).

### 3.2 Intermediate integration and latent spaces

To overcome the limitations of early concatenation, intermediate integration has emerged as a widely used and biologically informative paradigm for complex variant interpretation (Li M. et al., 2025). Instead of merging raw data, intermediate fusion architectures project each omics layer into a shared, lower-dimensional mathematical representation known as a latent space (Peng, 2025; Yinghua et al., 2025). By simultaneously learning the feature representations from multiple modalities during model training, algorithms can identify underlying biological structures that are fundamentally shared across different layers (Lan et al., 2024).

Techniques such as Multi-Omics Factor Analysis (MOFA) and deep representation learning leverage this strategy to decouple modality-specific noise from genuine cross-omics biological variance (Zhang D. et al., 2025; Bao et al., 2023). In the context of the variant-to-biomarker pipeline, intermediate integration excels at identifying composite signatures (Carvalho et al., 2026). For instance, a latent space model can learn that a specific unclassified structural variant (genomic layer) consistently co-occurs with a distinct profile of aberrant splicing (transcriptomic layer) and altered protein interaction interfaces (proteomic layer) (Walker et al., 2025). By unifying these signals in the latent space, intermediate integration can provide complementary functional evidence that contributes to VUS interpretation and supports the discovery of multi-dimensional biomarkers (Jeong et al., 2025).

### 3.3 Late integration and ensemble learning

Late integration, or decision-level fusion, adopts a fundamentally different architecture by processing each omics layer independently (Spooner et al., 2025). Separate machine learning models are trained exclusively on individual data modalities—for example, one model predicting pathogenicity based on genomic sequence context, and another predicting disease status based on transcriptomic dysregulation (Xin, 2025). The final predictive output is generated by combining the independent decisions of these modality-specific models using ensemble learning techniques, such as majority voting, weighted averaging, or meta-classification (Zhou et al., 2025).

A key advantage of late integration is its modularity, which can make it attractive for clinical applications where complete multi-omics profiles are not always available. In real-world diagnostic settings, acquiring complete multi-omics profiles for every patient is practically and economically challenging (Song Y. et al., 2026; Gouru, 2025). Late integration models are particularly well suited to missing data modalities because modality-specific predictors can operate independently; if proteomic data are unavailable for a particular patient, the ensemble can generate a prediction from the available genomic and transcriptomic models (Zhang W. et al., 2025). In practice, fusion strategies should explicitly account for modality availability—for example, by reweighting or aggregating only the predictions from available modalities—rather than requiring imputation of an entirely missing omics layer. This flexibility may facilitate the deployment of variant interpretation tools in heterogeneous clinical environments, although its practical performance and translation potential remain dependent on data quality, validation design, and implementation context (Ramakrishnaiah et al., 2025). In modern clinical workflows, patient data is naturally fragmented—a patient might have a targeted gene panel but lack a full transcriptomic profile. Late integration’s modularity can align well with electronic health record (EHR) systems, allowing clinicians to dynamically generate predictive scores based solely on the data at hand (Chen et al., 2026; Celik et al., 2025).

### 3.4 Network-based data fusion

Biological systems do not operate as isolated matrices but rather as highly interconnected interaction networks. Network-based integration strategies transform multi-omics data into topological graphs, where biological entities (variants, genes, proteins, metabolites) represent nodes, and their regulatory or physical interactions constitute the edges (Dipalma et al., 2025). This approach allows researchers to superimpose patient-specific multi-omics profiles onto established biological scaffolds, such as protein-protein interaction (PPI) networks or metabolic pathways (Lee D. et al., 2025).

By mapping a genetic variant onto a comprehensive interactome, computational algorithms can simulate how the functional consequence of that mutation propagates through the biological system (Shafi et al., 2026). Techniques such as random walk with restart or network diffusion can identify downstream functional modules that are significantly perturbed by the variant. This topology-driven fusion not only contextualizes the variant within a systemic framework but also provides a suitable representation for advanced Graph Neural Networks (GNNs), facilitating the discovery of network-level biomarkers that dictate disease pathophysiology (Mo et al., 2025; Yin et al., 2025).

### 3.5 Hybrid and hierarchical integration to bridge complexity and translation

As single-paradigm architectures reveal their intrinsic bottlenecks, hybrid or hierarchical integration has emerged as an increasingly used strategy for biomarker discovery (Hussein and Répás, 2026). This paradigm typically employs a multi-stage cascading architecture, often utilizing intermediate integration (such as autoencoders) to extract modality-specific latent features first, followed by a late-integration-style ensemble or a global attention mechanism for final clinical prediction (Daud et al., 2025).

Recent literature heavily emphasizes the use of multi-modal Transformers within this hybrid framework (Lin Y. et al., 2025). For example, contemporary studies published in bioinformatics journals frequently demonstrate how multi-omics attention networks can process heterogeneous inputs. The critical advantage here lies in the “Variant-Biomarker Link” (Liao et al., 2026). By leveraging cross-modal attention weights, these models can highlight genomic variants that contribute strongly to the model’s prediction and are associated with downstream transcriptomic abnormalities. However, attention weights and other attribution outputs indicate model-level importance rather than mechanistic or causal relationships; causal claims therefore require independent experimental or appropriately designed causal validation (Hu et al., 2025a; Stillwell, 2025). In my view, hybrid integration may offer a useful balance between the feature-extraction capabilities of early/intermediate fusion and the modularity of late fusion. However, its relative advantages depend on dataset characteristics, model design, validation strategy, and the intended clinical application. A comprehensive multidimensional analysis of these multi-omics integration paradigms is detailed in Table 2. By evaluating their core machine learning architectures, capacity to establish interpretable variant-biomarker links, tolerance to missing data, and overall clinical translation readiness, this table provides a methodological roadmap for translating complex biological data into deployable clinical tools.

**TABLE 2:** Multidimensional comparison of multi-omics integration strategies for clinical biomarker discovery.

Integration paradigm	Core mechanism	Representative ML architectures	Variant-biomarker link and interpretability	Tolerance to missing data	Clinical translation readiness	Key strength vs. Primary bottleneck	References
Early Integration (Data-level)	Concatenates raw omics features into a single unified matrix prior to model training	Standard Deep Neural Networks (DNNs), Random Forests, SVMs	Low: Massive, noisy feature spaces often drown out specific genetic variant signals with high-abundance transcriptomic data	Low: Requires strict sample overlap; missing modalities demand aggressive imputation	Low: High clinical deployment cost; requires all omics assays to be performed simultaneously for a single prediction	Strength: Captures holistic cross-modality interactions early Bottleneck: Severe “curse of dimensionality” (p≫n) causing model overfitting	(Peng, 2025)
Intermediate Integration (Latent-level)	Projects disparate omics matrices into a shared, lower-dimensional mathematical subspace	Variational Autoencoders (VAEs), Multi-Omics Factor Analysis (MOFA), Deep CCA.	Medium: Latent features are abstract; mandates *post hoc* Explainable AI tools (e.g., SHAP) to identify variants driving the biomarkers	Medium: Certain architectures (e.g., MOFA) can natively infer missing views/modalities	Medium: Requires complex preprocessing pipelines but offers better robustness to technical noise	Strength: Expertly distills shared biological variance from technical noise Bottleneck: High architectural complexity and hyperparameter tuning demands	(Huang et al., 2025)
Late Integration (Decision-level)	Trains modality-specific models independently, aggregating final predictions via ensembling	Ensemble methods (Majority Voting, Weighted Averaging, Meta-classifiers)	High (Modular): Easily traceable to see whether a genomic variant model or a proteomic model drove the final clinical decision	High: Highly modular; easily generates predictions even if an entire omics layer is missing	Potentially High: Modular structure may facilitate clinical deployment when complete multi-omics profiles are unavailable	Strength: Modular and flexible when omics modalities are incomplete or vary across patients. Bottleneck: May fail to capture complex synergistic interactions across omics layers	(Yang et al., 2026c)
Network-Based (Topological)	Superimposes patient profiles onto established biological scaffolds (e.g., PPI or metabolic graphs)	Graph Neural Networks (GNNs), Graph Convolutional Networks (GCNs), Random Walks	High: Can highlight associations between variants, biological pathways, and network hubs, but does not by itself establish mechanistic or causal relationships	Medium: Can tolerate missing node features, but sensitive to incomplete topological edges	Medium-High: Highly suitable for the development and validation of actionable, mechanistic biomarkers (e.g., drug targets)	Strength: Supports network-level contextualization of systemic biological perturbations Bottleneck: Heavily constrained by the incompleteness of existing biological databases	(Ray et al., 2025)
Hybrid/Hierarchical (New Paradigm)	Multi-stage cascading, typically extracting local modality features first before global integration	Hierarchical Neural Networks, Multi-modal Transformers	High: Attention mechanisms can highlight variant sites that contribute strongly to model predictions, without by themselves establishing causal effects	High: Combines the strengths of intermediate and late integration, offering multi-level fault tolerance	Medium: Clinical utility depends on architecture, dataset characteristics, and validation design	Strength: Can balance cross-modal feature learning with modular integration Bottleneck: Requires massive, well-annotated multi-omics datasets for training	(Jin et al., 2025)

## 4 Machine learning architectures

The successful implementation of the integration strategies discussed previously relies on selecting appropriate computational engines. The transition from a genetic variant to a robust clinical biomarker is rarely a linear problem; it involves navigating high-dimensional spaces, decoding complex sequence grammar, and mapping systemic biological networks. Consequently, the field has rapidly evolved from employing standard statistical models to deploying advanced deep learning architectures (Khan et al., 2025; Ajala et al., 2025). The current bottleneck in translational bioinformatics is no longer algorithmic power, but rather algorithmic appropriateness—matching the right machine learning paradigm to specific multi-omics data types and clinical objectives (Nazaretyan et al., 2025).

### 4.1 Classical machine learning algorithms

Before the widespread adoption of deep learning, classical machine learning algorithms established the foundation for variant interpretation and biomarker discovery. Tree-based ensemble methods, particularly Random Forests and Gradient Boosting Machines (e.g., XGBoost, LightGBM), remain widely used approaches for processing structured, tabular clinical and omics data (Anonto et al., 2025). These algorithms excel at handling heterogeneous features—seamlessly mixing categorical variant data with continuous expression or metabolite levels—making them well suited to early and late integration strategies (Namjoo et al., 2025). From a translational perspective, classical algorithms maintain a distinct advantage: interpretability. Recent literature heavily pairs XGBoost with *post hoc* Explainable AI (XAI) frameworks like SHAP (SHapley Additive exPlanations). This combination can help identify which genetic variants or clinical parameters contribute strongly to a model-derived risk score (Rafie et al., 2025; Siddiqua et al., 2025). While they struggle to extract raw spatial motifs directly from unaligned DNA sequences, classical algorithms serve as highly robust baselines and meta-classifiers in late-integration ensembles and are frequently used to develop integrated multi-omic risk models that incorporate polygenic risk information for complex traits (Kaipa et al., 2026).

### 4.2 Convolutional neural networks for sequences

Convolutional Neural Networks (CNNs), originally designed for image recognition, have revolutionized the analysis of linear biological sequences. In the genomic context, 1D-CNNs treat DNA or RNA sequences as one-dimensional spatial data. By sliding convolutional filters across the genome, these networks automatically learn to detect localized sequence motifs, such as transcription factor binding sites or splice consensus sequences, without prior human annotation (Wang and Liu, 2026; He et al., 2025). This architecture directly addresses the Variant of Uncertain Significance (VUS) challenge. Tools like SpliceAI utilize deep CNNs to predict how a single nucleotide variant—deeply intronic or exonic—will disrupt normal splicing machinery, translating a silent DNA mutation into a quantifiable transcriptomic aberration (Jaganathan et al., 2019). Similarly, frameworks like DeepSEA predict how non-coding variants alter chromatin accessibility and epigenetic marks (Zhou and Troyanskaya, 2015). However, contemporary research suggests that while CNNs are well suited to extracting highly localized regulatory grammar, they are increasingly being combined with Recurrent Neural Networks (RNNs) or Transformers to capture the broader genomic context required to link a static variant to a downstream molecular phenotype (Tabane et al., 2025).

### 4.3 Transformers and large genomic models

While CNNs excel at capturing local motifs, they often fail to capture long-range regulatory interactions, such as promoter-enhancer looping across thousands of base pairs. The introduction of the Transformer architecture, driven by its self-attention mechanism, has substantially improved the ability to model such long-range dependencies. Transformers evaluate the entire genomic sequence simultaneously, dynamically weighing the importance of distant genomic elements in regulating a specific focal variant (Kravchuk et al., 2025). This capability has catalyzed the era of genomic foundation models. Architectures like Enformer can predict multi-omic profiles (gene expression, chromatin states) directly from DNA sequences with high predictive accuracy, capturing regulatory dependencies across vast genomic distances (Avsec et al., 2021). In the protein domain, Transformer-based models like AlphaMissense have drastically improved the pathogenicity prediction of missense variants by learning the evolutionary “language” of protein sequences (Cheng et al., 2023). As an emerging trend, I anticipate that the future of clinical translation lies not in training these massive models from scratch, but in computationally lightweight fine-tuning (e.g., LoRA) of these foundation models on hospital-specific, multi-omics patient cohorts (Mojtahedi et al., 2026).

### 4.4 Autoencoders for signature discovery

When the clinical objective shifts from interpreting a single sequence to integrating massive, patient-level multi-omics profiles, autoencoders represent a widely used architecture. An autoencoder is an unsupervised neural network designed to compress high-dimensional input data into a lower-dimensional “bottleneck” (the latent space) and then reconstruct the original data. Variational Autoencoders (VAEs) are particularly powerful in multi-omics fusion. By feeding concatenated genomic, transcriptomic, and epigenetic matrices into a VAE, the network is forced to discard modality-specific noise and retain only the core, shared biological variance (Ru et al., 2025; Ko et al., 2025). Recent multi-omics oncology studies frequently employ VAEs to distill hidden prognostic signatures that single-omics analyses completely miss. By mining this compressed latent space, researchers can link a specific upstream variant to a holistic downstream molecular state, making Autoencoders widely used components of intermediate and hybrid integration strategies (Chang et al., 2025; Shi et al., 2025).

### 4.5 Graph neural networks for biological connectivity

Biological systems are inherently topological, making Graph Neural Networks (GNNs) a biologically informed architecture for variant-to-biomarker translation. Unlike CNNs or Transformers that process grids or sequences, GNNs operate directly on irregular graph structures, such as protein-protein interaction (PPI) networks, gene regulatory networks, or metabolic pathways (Zhang and Liu, 2025). When a genetic variant is identified, it acts as a perturbation at a specific node. GNNs utilize “message passing” to simulate how the functional consequence of this variant propagates through the network edges to neighboring proteins or genes (Dip and Zhang, 2026). A fascinating frontier in current literature is the application of GNNs in Spatial Transcriptomics (Spatial Omics), where cells themselves form the nodes of a physical graph (Kumar et al., 2026). By analyzing network topology, GNNs can identify critical “hub” biomarkers—downstream molecules whose network connectivity is severely disrupted by the variant. This approach shifts the translational focus from the isolated mutated gene to the dysregulated systemic module, bridging the final gap between genetic variation and clinical intervention (Qin et al., 2025). To bridge the gap between theoretical algorithmic concepts and practical clinical application, Table 3 provides a curated summary of state-of-the-art machine learning tools, categorizing them by their underlying architecture, accepted input modalities, and their direct contribution to variant interpretation frameworks (e.g., ACMG criteria) and multi-omics biomarker discovery.

**TABLE 3:** State-of-the-art machine learning tools for variant interpretation and multi-omics biomarker discovery.

Category	Tool/Model name	Primary architecture	Input modalities	Target biological output/Biomarker	Clinical utility and ACMG context	References
Sequence & Motif Decoders	SpliceAI	32-layer dilated residual CNN	Pre-mRNA/genomic sequence with 10-kb flanking context	Predicted splice-site usage and variant splice-impact scores	Computational evidence for potential splice-altering effects; may support variant prioritization and PP3/BP4 assessment when appropriately calibrated, but does not itself constitute PS3/BS3 functional evidence	(Jaganathan et al., 2019)
	DeepSEA	Convolutional neural network	Genomic DNA sequence	Predicted chromatin effects of sequence variants	Computational evidence for non-coding regulatory effects that may contribute to PP3/BP4 assessment; does not establish functional causality	(Zhou and Troyanskaya, 2015)
	PrimateAI-3D	Semi-supervised 3D convolutional neural network	Voxelized protein structural context and evolutionary information	Missense variant pathogenicity score	Computational prediction for missense variant prioritization; may contribute to PP3/BP4 assessment when appropriately calibrated	(Gao et al., 2023)
Genomic Large Language Models (LLMs)	AlphaMissense	AlphaFold-derived deep learning model	Protein sequence with evolutionary and structural context	Missense variant pathogenicity score	Computational pathogenicity prediction that may contribute to PP3/BP4 assessment when appropriately calibrated; it does not provide functional-assay evidence	(Cheng et al., 2023)
	Enformer	Convolutional encoder with Transformer/self-attention blocks	∼196-kb genomic DNA sequence, capturing regulatory interactions up to ∼100 kb	Gene-expression and epigenomic track predictions	Supports prediction and prioritization of non-coding regulatory variant effects; predictions require experimental validation for mechanistic or clinical interpretation	(Avsec et al., 2021)
Latent Space and Multi-Omics Fusion	MOFA+ (Multi-Omics Factor Analysis)	Bayesian factor-analysis framework with variational inference	Multi-modal omics data across samples or cells	Latent factors capturing major sources of variation	Unsupervised discovery of shared and modality-specific molecular variation for patient or cell stratification	(Argelaguet et al., 2020)
	OmiVAE	Variational Autoencoder (VAE)	Multi-omics continuous matrices (e.g., RNA-seq + DNA methylation)	Lower-dimensional latent features; Classification labels	Precision Oncology: Extracts highly compressed multi-omic signatures to predict tumor subtypes and clinical outcomes	(Zhang et al., 2019)
	scMDC	Deep Autoencoder with clustering	Single-cell multi-omics (scRNA-seq + scATAC-seq)	Single-cell multi-omic clusters/Cell-type specific signatures	High-Resolution Biomarkers: Identifies rare pathogenic cell subpopulations masked in bulk tissue analysis	(Lin et al., 2022)
Network & Graph Integrators	MOGONET	Graph Convolutional Network (GCN)	Multi-omics data + View-specific similarity graphs	Biomarker sub-networks; Disease classification scores	Biomarker Discovery: Identifies strongly connected, multi-omic functional modules driving disease pathology	(Wang et al., 2021)
	netDx	Patient Similarity Networks (PSN)	Any omics layers + clinical tabular data	Patient survival outcomes; Drug response predictions	Pharmacogenomics: Integrates diverse data to predict patient-specific therapeutic responses, enabling precise drug repositioning	(Pai et al., 2019)

## 5 Clinical translation and biomarker discovery

The ultimate success of any multi-omics machine learning model lies in its clinical utility. However, navigating the translational “valley of death”—the precarious gap between computational predictions and approved diagnostics—remains the most critical challenge of the variant-to-biomarker pipeline (Hsu et al., 2025; Lin M. et al., 2025). Importantly, biomarkers identified during model development or discovery should not be regarded as clinically validated biomarkers. Translation to clinical use requires independent replication in external cohorts, demonstration of technical reproducibility across assays and laboratories, and appropriate adjustment for relevant demographic, clinical, and technical confounders. Candidate biomarkers should also demonstrate incremental predictive or clinical value beyond established clinical variables and existing standard-of-care models before they can be considered ready for routine clinical implementation. By translating complex molecular data into candidate and clinically actionable biomarkers, AI frameworks are increasingly contributing to medical decision-making across the disease spectrum (Zhang et al., 2023).

### 5.1 Variant reclassification in rare diseases

In the realm of rare Mendelian disorders, the primary clinical bottleneck remains the interpretation of germline VUS. The ACMG/AMP variant classification guidelines demand rigorous evidence to assign pathogenicity, particularly functional evidence (PS3/BS3 criteria) (Lewis et al., 2025; Luo et al., 2025). Machine learning models integrating genomic and transcriptomic data can support this process by helping identify functionally relevant molecular abnormalities. Rather than relying solely on isolated *in vitro* assays, RNA-sequencing data analyzed with computational methods can provide evidence that a specific genomic variant is associated with aberrant splicing or monoallelic expression loss (Stark et al., 2025; Ren et al., 2026). Such evidence should be interpreted within ACMG/AMP criteria and relevant gene- or disease-specific specifications rather than being considered independently sufficient for PS3/BS3 assignment. As illustrated in Figure 4, this AI-driven multi-omics pipeline helps bridge the gap between static genomic data and dynamic functional evidence.

**FIGURE 4:**
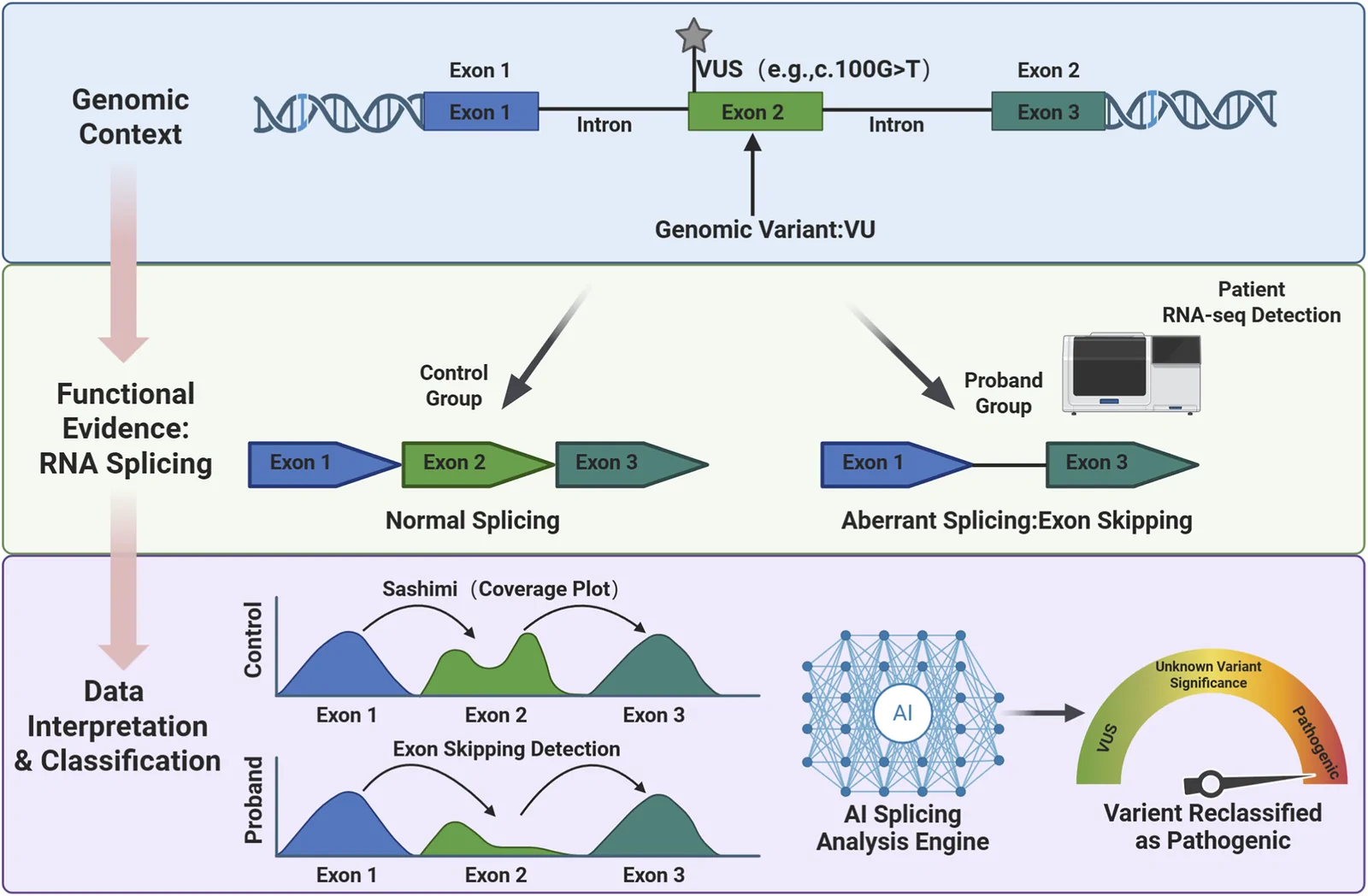
AI-driven reclassification of variants of uncertain significance (VUS) using integrated RNA-sequencing. The multi-omics pipeline illustrates how static genomic findings can be supplemented with functional evidence to support clinical variant interpretation. Top: A VUS is identified at the DNA level within a specific genomic context, lacking sufficient evidence for clinical classification. Middle: Transcriptomic analysis (RNA-seq) provides dynamic functional evidence by comparing the proband’s splicing events to a healthy control group. In this representative scenario, the VUS induces aberrant splicing (exon skipping). Bottom: Deep learning algorithms automatically extract and evaluate complex transcriptomic features, such as coverage alterations and novel splice junctions visualized via Sashimi plots. These transcriptomic findings provide functional evidence that can contribute to variant reclassification when integrated with other relevant evidence within an established clinical interpretation framework.

Recent landmark studies in cohorts of undiagnosed rare diseases demonstrate that integrating RNA-seq data can increase diagnostic yield by up to 10%–20% for cases previously stalled at the VUS level (Yépez et al., 2026). As sequencing costs continue to plummet and bioinformatic pipelines become standardized, RNA-guided variant interpretation is increasingly being evaluated for broader clinical use, although its implementation remains dependent on assay validation, standardized analytical pipelines, and integration with established variant-classification frameworks. By identifying these dynamic transcriptomic biomarkers, AI-assisted transcriptomic analyses can provide complementary functional evidence that contributes to VUS interpretation when integrated with genetic, clinical, segregation, and other relevant evidence within established classification frameworks. (Luo et al., 2025; De Cock et al., 2025).

### 5.2 Precision oncology and therapeutic targets

Cancer is fundamentally a disease of the genome, characterized by extreme mutational burden and profound cellular dysregulation. In precision oncology, identifying a somatic variant is only the first step; understanding its functional cascade is paramount for therapeutic intervention. Unlike germline VUS interpretation in Mendelian disease, somatic variant interpretation in oncology places greater emphasis on tumor-specific oncogenic significance, prognostic relevance, and therapeutic actionability. Machine learning models excel at integrating tumor genomics with deep proteomics—a field known as proteogenomics—to discover actionable biomarkers (Pevzner et al., 2025; Chen et al., 2025c). Studies from consortia such as the Clinical Proteomic Tumor Analysis Consortium (CPTAC) have shown that genomic alterations alone may incompletely predict protein abundance and phosphorylation states, which are the actual targets for most kinase inhibitors (Li et al., 2026b; Li J.-T. et al., 2026).

For example, neural networks can process integrated multi-omics profiles to predict which specific missense mutations will be translated into immunogenic neoantigens presented on the tumor cell surface. These algorithmically derived neoantigen biomarkers are critical for patient stratification in immune checkpoint inhibitor therapy and for the design of personalized mRNA cancer vaccines (Gschwind and Ossowski, 2025; Fan et al., 2025). Looking forward, I believe the integration of spatial multi-omics will be the next major frontier in this domain; to truly overcome tumor microenvironment-mediated resistance, models must learn not just what mutations the tumor harbors, but where those mutated cells are spatially located relative to the immune infiltrate. Consequently, multi-omics fusion transforms a simple catalog of tumor mutations into a dynamic, actionable map of therapeutic vulnerabilities (Li M.-Y. et al., 2025; Cheng et al., 2025).

### 5.3 Pharmacogenomics and drug repurposing

The efficacy and toxicity of pharmacological treatments exhibit vast inter-individual variability, largely driven by genetic variation in drug-metabolizing enzymes and drug targets. Pharmacogenomics seeks to identify predictive biomarkers that guide optimal drug selection and dosing (Vanderwerff et al., 2025). Machine learning accelerates this clinical translation by linking patient-specific genetic variants (e.g., in cytochrome P450 genes) to downstream transcriptomic and metabolomic phenotypes (Joseph et al., 2025).

Current bioinformatics literature is replete with network-based deep learning models that successfully map the off-target ripple effects of common drugs, highlighting novel repurposing candidates for rare genetic phenotypes. By training ensemble models on these integrated datasets, researchers can discover multi-omic signature biomarkers that accurately predict adverse drug reactions or therapeutic resistance before a drug is administered (Wu et al., 2025b; Liu et al., 2025). While these network strategies are computationally elegant, the real-world hurdle for multi-omic pharmacogenomics is highly regulatory. Approval bodies require robust, reproducible assays, meaning our AI pipelines must prioritize model stability and explainability over sheer predictive complexity when predicting severe adverse events. An additional challenge is population generalizability. Pharmacogenomic predictions may vary across ancestries because of differences in allele frequencies and linkage disequilibrium patterns, while many reference datasets remain disproportionately derived from a limited number of populations. Consequently, models developed or validated in one ancestry group may show reduced accuracy when transferred to other populations. The approaches reviewed here have not been uniformly evaluated across diverse ancestries, underscoring the need for ancestry-aware validation and external testing in population-diverse cohorts before clinical deployment. This computational approach highlights opportunities for drug repurposing, utilizing existing approved therapies to target newly identified multi-omic vulnerabilities in patient subpopulations (Zack et al., 2025; Sridharan and Sivaramakrishnan, 2026).

### 5.4 From polygenic risk scores to integrated multi-omic risk models

While rare variants drive Mendelian diseases, the genetic architecture of common, complex traits—such as cardiovascular disease, diabetes, and neurodegeneration—is highly polygenic. Traditional Polygenic Risk Scores (PRS) aggregate the subtle effects of millions of genomic variants, but they suffer from limited portability across diverse ancestries and fail to capture dynamic environmental influences (Woerner et al., 2025; Feng et al., 2026). Recent large-scale biobank studies have proven that incorporating functional molecular weights (e.g., via Transcriptome-Wide Association Studies, TWAS) significantly improves both the mechanistic interpretability and the cross-ancestry transferability of these scores (Liu et al., 2026).

Machine learning is increasingly being used to extend conventional PRS by integrating genetically derived risk with functional molecular information, such as eQTLs, pQTLs, transcriptomic profiles, proteomic markers, and metabolomic features (Li Q. et al., 2026). Importantly, conventional PRS remain genetically derived and essentially time invariant, whereas models incorporating dynamic molecular measurements are more appropriately described as integrated multi-omic risk models (Guo et al., 2025). These models may provide complementary information on current biological state and disease trajectory beyond inherited genetic susceptibility, although their added predictive value requires rigorous external validation (Kioroglou et al., 2025). To synthesize the diverse translational applications discussed above, Table 4 provides a comprehensive overview of representative clinical scenarios across multiple medical domains, illustrating how machine learning-driven multi-omics integration effectively translates specific variant contexts into actionable clinical biomarkers.

**TABLE 4:** Representative applications and translational status of ML-integrated multi-omics biomarkers.

Clinical domain	Disease type	Variant context	Integrated omics layers	AI-derived biomarker	Potential clinical utility/Translational status	References
Rare Mendelian Diseases	Neurodevelopmental Disorders	*De novo* missense VUS	Genomics + Epigenomics	Disease-specific DNA methylation signature (EpiSign)	Exploratory/Candidate: May provide supportive evidence for VUS interpretation when integrated with established classification criteria	(Mascheroni et al., 2025)
	Muscular Dystrophies (DMD)	Deep intronic VUS	Genomics + Transcriptomics	Cryptic splicing and pseudoexon activation signature	Exploratory/Candidate: May contribute to VUS reclassification and inform potential ASO-based therapeutic strategies	(Li et al., 2026e)
	Mitochondrial Disorders	Nuclear/mtDNA VUS	Genomics + Proteomics + Metabolomics	OXPHOS complex defect and distinct acylcarnitine profile	Exploratory/Candidate: May provide supportive biochemical evidence for variant interpretation and diagnostic evaluation	(Zhang et al., 2025d)
	Cardiomyopathies (HCM/DCM)	Splice-site/Frameshift VUS	Genomics + RNA-seq (Blood)	Allele-specific expression (ASE) indicating NMD	Exploratory/Candidate: May provide functional evidence of haploinsufficiency and support subsequent clinical evaluation	(Lindholm et al., 2025)
	Inborn Errors of Metabolism	Biallelic missense VUS	Genomics + Metabolomics	Toxic intermediate accumulation signature	Exploratory/Candidate: May support identification of functional enzyme deficiency and inform clinical management	(Xie et al., 2025)
	Cystic Fibrosis (CF)	CFTR folding/trafficking VUS	Genomics + Proteomics	Intracellular protein mislocalization signature	Exploratory/Candidate: May support patient stratification for CFTR-targeted therapies	(Pankow et al., 2025)
	Primary Immunodeficiencies	Immune-related gene VUS	Genomics + scRNA-seq	Aberrant immune cell subpopulation clustering	Exploratory/Candidate: May identify disease-relevant cellular abnormalities and inform further therapeutic evaluation	(Gan et al., 2025)
Precision Oncology	Non-Small Cell Lung Cancer	Somatic EGFR/ALK variants	Genomics + Transcriptomics	Tyrosine kinase inhibitor (TKI) resistance signature	Exploratory/Candidate: May identify resistance-associated signatures and inform subsequent therapeutic evaluation	(Kuribayashi et al., 2026)
	Melanoma	Somatic BRAF/NRAS variants	Genomics + Spatial Transcriptomics	Tumor microenvironment (TME) immune exclusion state	Exploratory/Candidate: May help predict immunotherapy response and identify candidates for combination strategies	(Yang et al., 2025b)
	Breast/Ovarian Cancer	Germline/Somatic BRCA1/2 VUS	Genomics + Transcriptomics	Homologous Recombination Deficiency (HRD) signature	Exploratory/Candidate: May contribute to characterization of HRD-associated states and therapeutic stratification	(Wang et al., 2025b)
	Colorectal Cancer	Somatic MMR variants	Genomics + Epigenomics (Methylation)	MSI-High/CpG Island Methylator Phenotype (CIMP)	Exploratory/Candidate: May support molecular stratification relevant to immunotherapy response	(Zhang et al., 2025e)
	Pediatric B-ALL (Leukemia)	Structural variants/Fusions	Genomics + Transcriptomics + Proteomics	Aberrant kinase activation network signature	Exploratory/Candidate: May identify kinase-activation signatures and potential targeted therapeutic opportunities	(Pushel et al., 2026)
	Glioblastoma (GBM)	Somatic IDH1/2 variants	Genomics + Epigenomics + Metabolomics	Hypermethylation and 2-Hydroxyglutarate (2-HG) oncometabolite profile	Exploratory/Candidate: May support molecular subtyping and prognostic stratification	(Xu et al., 2025)
	Prostate Cancer	Androgen Receptor (AR) variants	Genomics + Proteomics	Splice variant (e.g., AR-V7) protein expression signature	Exploratory/Candidate: May support prediction of treatment resistance and therapeutic stratification	(Xu et al., 2026a)
	Clear Cell Renal Cell Carcinoma (ccRCC)	Somatic VHL/BAP1/PBRM1 variants	Genomics + Transcriptomics + Epigenomics	Angiogenic vs. Immune-infiltrated multi-omic tumor state	Exploratory/Candidate: May characterize angiogenic and immune tumor states relevant to treatment selection	(Xu et al., 2026b)
Pharmacogenomics (PGx)	Cardiovascular (Clopidogrel)	CYP2C19 SNPs	Genomics + Metabolomics	Poor metabolizer/Inactive active-metabolite profile	Established PGx context; multi-omic extension remains investigational: May refine prediction of antiplatelet response beyond CYP2C19 genotype alone	(Piccolotto et al., 2026)
	Oncology (Fluorouracil/5-FU)	DPYD variants	Genomics + Proteomics	DPD enzyme functional deficiency signature	Established PGx context; multi-omic extension remains investigational: May complement DPYD-based risk assessment for fluoropyrimidine toxicity	(Rodríguez-García et al., 2026)
	Psychiatry (SSRIs)	CYP2D6/CYP2C19 SNPs	Genomics + Transcriptomics	Altered neurotransmitter pathway network	Emerging/Candidate: May complement CYP2D6/CYP2C19 information for antidepressant response stratification	(Maillard et al., 2026)
	Gastroenterology (Thiopurines)	TPMT/NUDT15 SNPs	Genomics + Metabolomics	Thiopurine cytotoxic metabolite accumulation	Established PGx context; multi-omic extension remains investigational: May complement TPMT/NUDT15-based assessment of thiopurine toxicity	(Zhu et al., 2025)
Complex Traits (Integrated Multi-Omic Risk Models)	Coronary Artery Disease (CAD)	Genome-wide polygenic SNPs	Genomics + Metabolomics + Proteomics	Integrated genetic–molecular risk signature	Exploratory/Candidate: May improve cardiovascular risk stratification beyond conventional genetic and clinical predictors	(Ratman et al., 2025)
	Type 2 Diabetes (T2D)	Genome-wide polygenic SNPs	Genomics + Transcriptomics (eQTLs)	Islet cell dysfunction and insulin resistance signature	Exploratory/Candidate: May improve risk stratification by integrating inherited genetic risk with functional molecular information	(Hu et al., 2025b)
	Alzheimer’s Disease (AD)	APOE + Polygenic SNPs	Genomics + Epigenomics + Proteomics	Neuro-inflammatory and early amyloid cascade signature	Exploratory/Candidate: May support earlier risk stratification and research-based patient selection	(Beydoun et al., 2025)
	Rheumatoid Arthritis (RA)	HLA + Genome-wide SNPs	Genomics + Single-cell Transcriptomics	Synovial macrophage hyperactivation signature	Exploratory/Candidate: May support molecular stratification relevant to biologic treatment response	(Binvignat et al., 2024)

## 6 Challenges and future perspectives

Despite the transformative potential of machine learning in bridging the genotype-phenotype gap, the seamless integration of multi-omics data into routine clinical workflows remains a work in progress. Translating a computational variant-to-biomarker pipeline from a controlled research environment to robust bedside applications requires overcoming several formidable technical, analytical, and translational hurdles. Addressing these challenges will define the next decade of genomic medicine.

### 6.1 Explainable AI in clinical practice

Despite the transformative potential of machine learning in bridging the genotype-phenotype gap, translating computational variant-to-biomarker pipelines into routine clinical workflows requires overcoming significant translational hurdles, foremost among them being the opaque, “black-box” nature of advanced deep learning architectures. While models such as Convolutional and Graph Neural Networks have demonstrated strong predictive performance in variant pathogenicity prediction, they rarely articulate their underlying biological rationale. In clinical genetics, accuracy alone is insufficient; algorithmic transparency is a regulatory and ethical mandate, as physicians cannot confidently alter a patient’s therapeutic regimen or reclassify a VUS based on an uninterpretable algorithmic score.

Beyond discrimination accuracy and interpretability, clinical deployment also requires well-calibrated predictive probabilities and reliable uncertainty estimates. Calibration should therefore be evaluated using tools such as calibration curves and Brier scores, while confidence intervals can quantify the stability of model performance across resampled or external cohorts. This is particularly important for variant classification, VUS interpretation, pharmacogenomic recommendations, and biomarker discovery, where overconfident predictions may directly affect clinical decisions. Models should also incorporate mechanisms for detecting out-of-distribution inputs and, when predictive uncertainty is unacceptably high, be allowed to abstain from making a definitive prediction and instead defer the case for further testing or expert review.

Explainable AI (XAI) techniques, such as SHAP and attention visualization, can improve model transparency by identifying features that contribute strongly to a prediction. However, these attribution outputs reflect model-level associations and should not be interpreted as definitive biological, mechanistic, or causal explanations. Their clinical interpretation therefore requires integration with independent biological evidence and established variant-interpretation frameworks.

### 6.2 Data heterogeneity and standardization

Compounding this interpretability challenge is the pervasive heterogeneity and fragmentation of real-world biological datasets. Genomic, transcriptomic, and proteomic data are frequently generated across disparate sequencing platforms and clinical centers, resulting in severe technical noise, batch effects, and a lack of unified clinical ontologies that hamper cross-institutional data pooling. Such batch effects can arise from differences in sample collection and handling, sequencing platforms, laboratory protocols, and cohort recruitment, and may cause machine learning models to capture site- or protocol-specific signatures rather than true biological variation. Although batch-correction and harmonization procedures are therefore essential, overly aggressive correction may also remove biologically meaningful signals when technical factors are correlated with genuine disease- or population-related differences. Accordingly, batch correction should be carefully evaluated to reduce technical variation while preserving relevant biological structure. Addressing this heterogeneity therefore requires rigorous and biologically informed standardization pipelines, guided by global initiatives such as those from the Global Alliance for Genomics and Health (GA4GH).

Equally important is leakage-aware model validation. All data-dependent preprocessing—including normalization, imputation, dimensionality reduction, and feature selection—should be learned exclusively from the training folds, while hyperparameter optimization should be performed using nested cross-validation or an independent validation set rather than the held-out test data. Data partitioning should also occur at the patient level, ensuring that repeated samples from the same individual do not appear across training and validation/test sets. Where cohorts include related individuals, or where study- or center-specific effects could induce dependency or batch-related leakage, group-aware splitting should also be considered to avoid inflated estimates of model generalizability.

Beyond internal validation, multi-omics models should be externally evaluated across independent institutions and diverse populations, including ancestry groups, while accounting for realistic patterns of missing omics modalities. Prospective clinical validation is ultimately required to determine whether calibrated predictions remain generalizable and clinically useful in real-world workflows.

Furthermore, strict data privacy regulations often prevent the centralized aggregation of sensitive patient multi-omics profiles. To circumvent this barrier, the deployment of Federated Learning architectures is emerging as a critical solution. Federated learning allows robust machine learning models to be trained collaboratively across decentralized hospital networks without ever moving the raw data, thereby preserving patient privacy while achieving the massive statistical power necessary for rare variant interpretation and biomarker discovery.

### 6.3 Single-cell and spatial omics

Looking beyond current analytical and infrastructural limitations, the future resolution of biomarker discovery is poised for a paradigm shift driven by single-cell and highly multiplexed spatial omics technologies. Historically, multi-omics integration has relied heavily on “bulk” tissue sequencing, which calculates the average molecular signal across millions of heterogeneous cells, inadvertently masking critical rare-cell populations and tissue-specific regulatory dynamics. By mapping genetic variants to multi-omic phenotypes at single-cell resolution while preserving the native architectural coordinates of the tissue, spatial omics provide rich functional context. As advanced spatial neural networks mature, they will be able to decode exactly how a specific genetic alteration perturbs its surrounding multi-omic microenvironment—such as disrupting immune cell infiltration within a tumor. Integrating these ultra-high-resolution layers into the variant-to-biomarker pipeline will enable the discovery of highly specific, spatiotemporal biomarkers, ultimately realizing the full promise of precision medicine.

## 7 Conclusion

Bridging the genotype-phenotype gap necessitates a paradigm shift from static genomic observation to integrative functional interpretation. Multi-omics integration provides valuable biological context that can support VUS interpretation, improve the characterization of complex disease traits, and inform the prediction of differential drug responses. As demonstrated, machine learning serves as the essential computational scaffold, capable of distilling high-dimensional biological noise into predictive pharmacogenomic and diagnostic signatures. However, the path to seamless clinical implementation remains obstructed by data siloization, algorithmic opacity, and the economic burden of comprehensive profiling. Our analysis suggests that future breakthroughs will not stem from sheer algorithmic complexity alone, but from “pharmacogenomic and clinical translation readiness”. Priority must be given to missing-data-tolerant modular architectures, multi-modal foundation models, explainable AI for regulatory compliance, and standardized benchmarking across diverse longitudinal cohorts. While not a panacea, the convergence of biologically-constrained AI and high-throughput omics offers a pragmatic framework to transition precision pharmacotherapy from a theoretical ideal into a multidimensional clinical reality.
